# Prevalence of Patent Foramen Ovale in Patients with Non-Obstructive Coronary Artery Disease (PROVA) Study

**DOI:** 10.3390/jcdd12040108

**Published:** 2025-03-21

**Authors:** Abdelhak el Bouziani, Lars S. Witte, Rutger G. T. Feenstra, Mick P. L. Renkens, Janneke Woudstra, Jan G. P. Tijssen, Arja S. Vink, Yolande Appelman, Maik J. D. Grundeken, Bart Straver, Jan J. Piek, Berto J. Bouma, Robbert J. de Winter, Marcel A. M. Beijk

**Affiliations:** 1 Department of Cardiology, Heart Center, Amsterdam UMC, Location Academic Medical Center, University of Amsterdam, Meibergdreef 9, 1105 AZ Amsterdam, Noord-Holland, The Netherlands; 2 Department of Cardiology, Heart Center, Amsterdam UMC, Location VU Medical Center, VU University, De Boelelaan 1117, 1081 HV Amsterdam, Noord-Holland, The Netherlands; 3 Department of Pediatric Cardiology, Heart Center, Amsterdam UMC, Location Academic Medical Center, University of Amsterdam, Meibergdreef 9, 1105 AZ Amsterdam, Noord-Holland, The Netherlands

**Keywords:** angina pectoris, patent foramen ovale, angina pectoris with no obstructive coronary artery disease, migraine with aura, right-to-left shunt, coronary artery spasm

## Abstract

(1) Background: Prevalence of patent foramen ovale (PFO) in the general population is estimated at around 24%. We hypothesized that right-to-left shunting (RLS) resulting from PFO might contribute to angina symptoms in patients with coronary artery spasm (CAS), potentially triggered by vasoactive metabolites. Therefore, the aim of this study was to investigate the prevalence of PFO-related RLS in patients with documented CAS. (2) Methods: This single-center prospective cohort study included patients with documented CAS undergoing transthoracic echocardiography (TTE), including a contrast bubble study between 2021 and 2023. The Seattle Angina Questionnaire (SAQ) and Migraine Disability Assessment (MIDAS) were used to survey patients. (3) Results: RLS (PFO group) was observed in 11 of the 48 patients included (23%). In the PFO group, 64% had epicardial spasm and 36% microvascular spasm. Furthermore, RLS was more prevalent in patients with CAS and concomitant migraine (29%). Remarkably, the density plot of the SAQ summary score showed a worse score for patients with RLS (median of 38 [Q1–Q3: 31–49]) than patients without RLS (median of 49 [Q1–Q3: 41–55]) (*p* = 0.0282). (4) Conclusions: The prevalence of RLS due to PFO in patients with CAS was in line with the PFO prevalence in the general population, and patients with RLS are more symptomatic according to the SAQ summary score. Whether PFO closure could be beneficial to patients with CAS and concomitant migraine requires further investigation.

## 1. Introduction

Patent foramen ovale (PFO) is a septal defect between the right and left atrium through which right-to-left shunting (RLS) may occur at rest or under certain circumstances such as temporary pressure-elevating moments (i.e., postural changes, sneezing, diving or Valsalva maneuvers). The prevalence of PFO in the general population is estimated at approximately 20–25%, and the opening of the PFO tunnel may vary substantially between subjects, with a large PFO of >10 mm in diameter present in 1% of the population [1,2]. Although most patients remain asymptomatic throughout their lives, the presence of a PFO is associated with certain types of diseases, e.g., paradoxical embolism, migraine with aura, decompression illness, and arterial deoxygenation syndromes.

Theoretically, a PFO with a RLS may allow for the transit of metabolites from the venous circulation (i.e., right atrium) directly to the arterial circulation (i.e., left atrium) which may cause a pathophysiological effect, for example, in the heart and/or brain. Coronary artery spasm (CAS) is a frequent mechanism of angina in patients with non-obstructed coronary arteries (ANOCA); however, the pathophysiology has not been completely elucidated, nor has the association with RLS [3,4]. It has been suggested that RLS may play a role as a trigger due to the shunting of vasoactive metabolites for the occurrence of migraine headaches; therefore, given the association between ANOCA and migraine, it might also be a trigger for episodes of angina in patients with coronary vasomotor disorder [5,6,7]. Recently, studies have shown that women with ANOCA have a higher prevalence of migraine compared to the general population (50% vs. 10%, respectively) [5]. Currently, the association between PFO and coronary vasomotor dysfunction is unknown. The aim of this study was to investigate the prevalence of PFO, using transthoracic echocardiography with bubble contrast in patients with CAS documented with an intracoronary function testing (ICFT).

## 2. Materials and Methods

### 2.1. Study Design

This single-center prospective cohort study included patients with typical and atypical angina pectoris in the absence of obstructive epicardial coronary artery disease on invasive coronary angiography who were referred as 2nd and 3rd opinions to the tertiary ANOCA clinic of the Amsterdam UMC (Amsterdam, The Netherlands). Amsterdam UMC is a tertiary center for patients with ANOCA. All patients underwent clinically indicated ICFT to establish the diagnosis CAS and the specific endotype. ICFT was conducted by dedicated interventional cardiologists who also correlate the results with clinical findings and use them to guide patient treatment. Hereafter, ANOCA patients were selected and invited to participate in the PROVA study between 2021 and 2023. Patients with a life expectancy below one year, an active infection requiring therapy, or an inability to provide written informed consent were excluded.

### 2.2. Coronary Angiography and ICFT

All patients underwent a diagnostic coronary angiography to exclude significant obstructive coronary artery disease using routine techniques. Prior to the ICFT, beta blockers were discontinued for at least 72 h, and all other vasoactive medication for at least 48 h. ICFT was performed with the use of a pressure and flow (ComboWire XT, Philips-Volcano, San Diego, CA, USA) predominantly in the left coronary artery for continuous measurement of the average peak velocity (APV). Aortic pressure (Pa) was continuously registered through the guiding catheter. CAS was tested by means of acetylcholine (ACh) spasm provocation, consisting of manual injection ACh within 1 min and incremental ACh doses of 2, 20, 100, and 200 µg. Incremental ACh injections were halted when epicardial vasospasm (>90% lumen narrowing) was documented. Coronary angiography was performed after each incremental dose in similar projections. At the end of the ACh testing, intracoronary nitroglycerine (200 µg) was administered. In our center, coronary vasoreactivity testing starts with the left coronary artery, and when found negative, the right coronary artery is tested with a single dose of 80 µg ACh. A surface electrocardiogram (ECG) and all hemodynamic data were recorded on a dedicated console (ComboMap, Volcano Corporation, San Diego, CA, USA), and the 12-lead ECG was continuously recorded using Mac-Lab (GE, USA) [8,9].

CAS was diagnosed according to the diagnostic criteria used by the Coronary Vasomotor Disorders International Study Group (COVADIS) working group [10]. Epicardial coronary spasm was diagnosed when ACh induced (i) recognizable angina; (ii) ECG changes suggestive of ischemia, which included ST-deviation of 0.1 mV in at least two contiguous leads; and (iii) >90% epicardial vasoconstriction by visual assessment at coronary angiography. Microvascular coronary spasm was diagnosed when the first two criteria mentioned above were met in absence of an epicardial vasoconstriction of >90% [11].

### 2.3. Echocardiographic Assessment

All patients underwent a complete transthoracic echocardiography (TTE) (Vivid E95 Ultrasound System, GE Healthcare, Horten, Norway) assessment, including agitated saline injection to investigate whether PFO was present. RLS was semi quantified graded (grade 0 = no microbubbles; grade I = < 5 microbubbles (mild shunting); grade II = 5–25 microbubbles (moderate shunting); grade III = > 25 microbubbles (large shunting); and grade IV = opacification of the entire left atrium) [12]. The RLS was assessed within the first four cardiac cycles after opacification of the right atrium. If there was no RLS observed, the injection with agitated saline was repeated with the patient performing the Valsalva maneuver.

### 2.4. Patient-Reported Questionnaires

The Seattle Angina Questionnaire (SAQ) and the Migraine Disability Assessment questionnaire (MIDAS) are both validated instruments and were used to survey patients at inclusion. Furthermore, patients reported whether they have migraine with aura symptoms. SAQ reports on physical limitation (subdomain A), angina stability (subdomain B), angina frequency (subdomain C), treatment satisfaction (subdomain D), and quality of life (QoL) (subdomain E). The results of the SAQ were summarized as the summary score including the subdomains A, C, and E. A score of 0 to 24 represents a poor health status, 25 to 49 a fair health status, 50 to 74 a good health status, and 75 to 100 an excellent health status [13].

MIDAS Questionnaire quantifies the impact of migraine on patients’ daily life and functioning. This is a four-point grading system: grade I (0–5 points, little or no disability), grade II (6–10 points, mild disability); grade III (11–20 points, moderate disability); and grade IV (21 or greater, severe disability) [14].

### 2.5. Statistical Analysis

Categorical variables were presented as frequencies and percentages. Continuous variables were summarized as mean ± standard deviation (SD) if they were normally distributed. Otherwise, median and 95% confidence interval (CI) or interquartile range (IQR) were used for non-normal distribution. The Shapiro–Wilk test was performed to determine data distribution. The Wilcoxon rank sum test, Fisher’s exact test, and Pearson’s Chi-squared test were executed in cases where it was necessary of test for differences between two independent groups. The Kolmogorov–Smirnov (K-S) test was utilized to test for differences in density plots. As this is considered a pilot study, power analysis was not conducted. Statistical significance was defined as a *p*-value < 0.05. Analyses were performed with SPSS 28.0.0.1 (IBM corporation, Armonk, New York, NY, USA) and R studio software version 4.3.2, Boston, MA, USA.

## 3. Results

### 3.1. Characteristics of the Study Population

A total of 50 ANOCA patients were screened for inclusion. Two patients were excluded due to incomplete ICFT. Forty-eight patients with a positive ICFT were included in this study. The baseline characteristics are shown in Table 1. Out of the 48 patients with CAS included in the study, 11 patients (23%) had a RLS due to a PFO, as observed with TTE and agitated saline (PFO group); and 37 patients (77%) did not have a RLS (non-PFO group). The mean age of the overall study population was 58 ± 10 years; for the PFO group, it was 54 ± 11 years; and for the non-PFO group, it was 60 ± 9 years (*p* = 0.154). The baseline characteristics between the two groups were not different except for the body mass index (*p* = 0.015). In the PFO group, 7 out of the 11 patients (64%) suffered from migraines compared to 17 out of 37 patients (46%) in the non-PFO group (*p* = 0.303). Furthermore, RLS was numerically more prevalent in patients with CAS and migraine (7/24 = 29%) compared to patients without migraine (4/24 = 17%). Overall, 32 patients (67%) were diagnosed with epicardial spasm and 16 patients (33%) with microvascular spasm. In the PFO group, seven patients (64%) had epicardial spasm and four patients (36%) had microvascular spasm. In the non-PFO group, 25 patients (68%) had epicardial spasm and 12 patients (32%) had microvascular spasm. The majority of patients had either epicardial spasm of the left anterior descending (LAD) alone or multivessel spasm of the LAD and circumflex artery (Cx). Importantly, none of the patients with PFO had significant epicardial obstructive coronary artery disease. However, one patient received a percutaneous coronary intervention due to iatrogenic occlusive coronary dissection due to wiring during the ICFT.

### 3.2. Echocardiographic Outcomes

The echocardiographic characteristics are summarized in Table 2. In eight patients, RLS occurred only after the Valsalva maneuver. One patient had grade I, five patients had grade II, and five patients had grade III RLS. Opacification (grade IV RLS) of the left atrium and/or ventricle was not observed (Table 2). Only two patients of the PFO group had an atrial septum aneurysm (ASA). During the echocardiographic assessment, 96% of patients had a normal left ventricular function, and left ventricular ejection faction (LVEF) was not different between the PFO and non-PFO group (*p* = 0.162). There was no difference in left ventricle diastolic function (PFO-group: 100% normal diastolic function vs. non-PFO group 92% normal diastolic function; *p* = 1.00). Severe valvular disease was not observed in any of the patients.

### 3.3. Outcomes of the Patient-Reported Questionnaires

**Figure 1:**
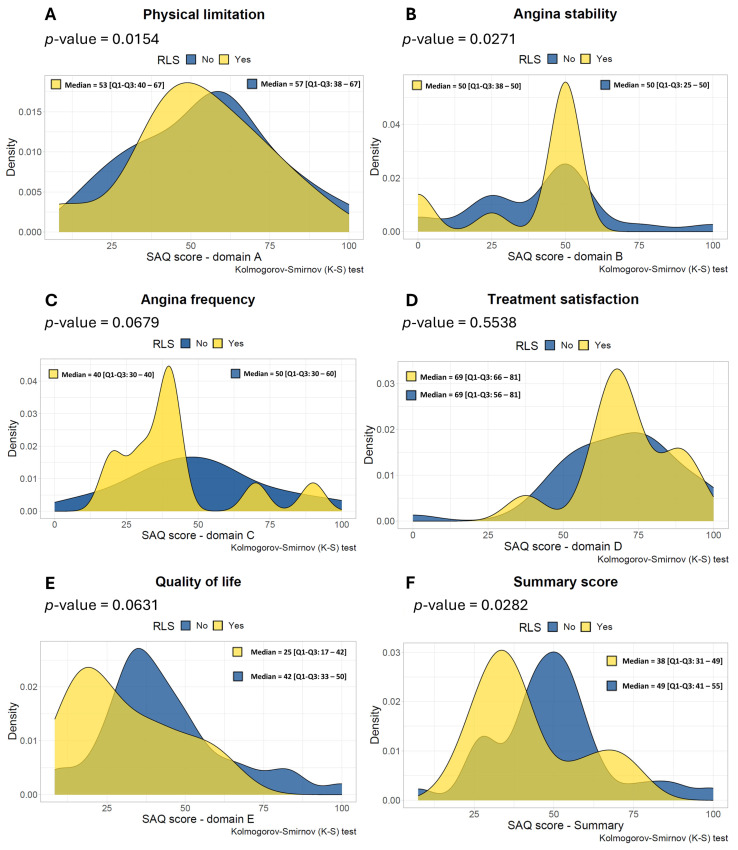
Density plots of the Seattle Angina Questionnaire scores across different subdomains. This figure consists of the subdomains (**A**–**E**) and summary score of all included patients (*n* = 48), whereas the yellow color represents patients with RLS due to a Patent Foramen Ovale (*n* = 11) and the blue color represents patients without RLS (*n* = 37). (**A**)—physical limitation, (**B**)—angina stability, (**C**)—angina frequency, (**D**)—treatment satisfaction and (**E**)—quality of life and the SAQ summary score (**F**). RLS = right-to-left shunting. SAQ = Seattle Angina Questionnaire.

The distribution of the SAQ scores of the subdomains (A to E), as well as the SAQ summary score in multiple density plots, are shown in Figure 1. The median of the SAQ summary score at baseline was 38 [Q1–Q3: 31–49] for the PFO group and 49 [Q1–Q3: 41–55] for the non-PFO group. The SAQ summary score was significantly different between the PFO group and the non-PFO group (*p* = 0.0282) (Figure 1F) as there was a trend for both subdomains C and E (*p* = 0.0679 and *p* = 0.0631 life, respectively) (Figure 1C,E). Subdomains A, B, and D did not show any difference (*p* = 0.9154, *p* = 0.8271, and *p* = 0.5538, respectively).

All patients filled out the MIDAS Questionnaire at baseline regardless of the presence of migraine. The results of the patient-reported symptoms according to the MIDAS Questionnaires are presented in Table 3. The median MIDAS score of all patients (*n* = 48) at baseline was 0 [Q1–Q3: 0–10]; in the PFO group, it was 2 [Q1–Q3: 0–12]; and in the non-PFO group, it was 0 [Q1–Q3: 0–9] (*p* = 0.368). In patients with migraine (*n* = 24), the median MIDAS score at baseline in the PFO group was 12 [Q1–Q3: 2–19]; and in the non-PFO group, it was 9 [Q1–Q3: 0–16] (*p* = 0.631). Of the seven patients with migraine and an RLS due to PFO, five patients had epicardial vasospasm and two patients had microvascular spasm. In addition, one patient had a grade I RLS, four patients had a grade II RLS, and two patients had a grade III RLS. In patients with migraine and aura (*n* = 15), no significant difference was observed in the MIDAS score between the PFO group and the non-PFO group (*p* = 1.000).

**Figure 2:**
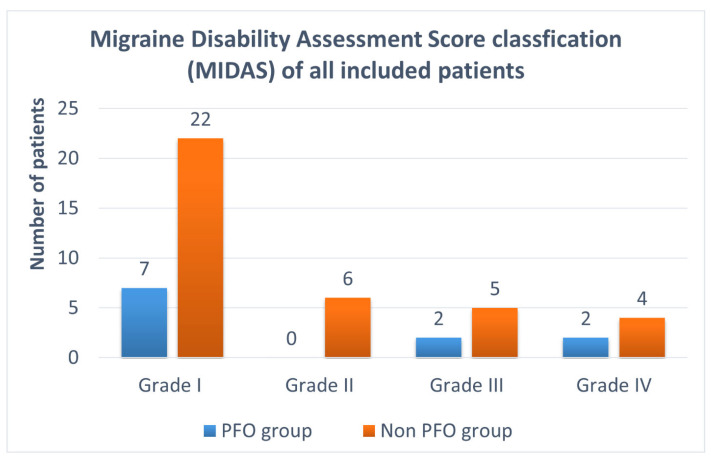
MIDAS classification of all included patients: 11 patients with a PFO and 37 patients without a PFO. Grade I (= 0–5 points, little or no disability); grade II (= 6–10 points, mild disability); grade III (= 11–20 points, moderate disability); and grade IV (= 21 or greater, severe disability). MIDAS = Migraine Disability Assessment Questionnaire; PFO = patent foramen ovale.

The MIDAS classification is illustrated in Figure 2. Seven patients of the PFO group were classified as grade I—little or no disability (64%); two patients as grade III—moderate disability (18%); and two patients as grade IV—severe disability (18%). There were no patients with grade II—mild disability due to migraine in the PFO group. In the non-PFO group, 22 patients were classified as grade I (59%), six patients as grade II (16%), five patients as grade III (14%), and four patients as grade IV (11%).

## 4. Discussion

To the best of our knowledge, this is the first study to investigate the prevalence of PFO-related RLS demonstrated with transthoracic contrast echocardiography in patients with CAS. The main findings of our study are as follows: (a) RLS prevalence due to PFO in patients with epicardial or microvascular spasm documented with an ICFT was in line with the prevalence of the general population; (b) RLS was more prevalent in patients with both epicardial or microvascular spasm documented with an ICFT and migraine; and (c) patients with RLS and epicardial or microvascular spasm are more symptomatic according to the SAQ summary score. Furthermore, we found that BMI was significantly higher in the non-PFO group (*p* = 0.015). However, it seems that this is a coincidental finding as we have no evidence that BMI has an effect on the presence of PFO.

The prevalence of RLS due to a PFO in patients with epicardial or microvascular spasm documented with an ICFT was 23% in our study. This corresponds with the overall prevalence of PFO in the general population determined in the autopsy study of Hagen et al. [1]. In this study, in 965 autopsy specimens of human hearts, the prevalence of a PFO was 27.3% and did not differ between male and female subjects. However, the prevalence of a PFO progressively declined with increasing age, with the results showing 34.3% in subjects younger than 30 years, 25.4% in subjects between 30 and 80 years, and 20.2% in subjects between 80 and 100 years [1]. Moreover, in a study by Tang et al., the prevalence of a PFO in a large Chinese population of urban residents over 20 years was 23.4% [15].

Previously, we have shown that there is no correlation between the grade of RLS measured by transthoracic echocardiography contrast bubble study using agitated saline and the anatomical PFO orifice area measured by transesophageal echocardiography [16]. Although the majority of patients had a grade II (5–25 microbubbles) or III (> 25 microbubbles) RLS (which corresponds with a moderate or large shunt), in 8 out of 11 patients, RLS was only observed after the Valsalva maneuver; therefore, the true size of the PFO in our study population remains unknown. Whether CAS may be triggered by vasoactive metabolites that transit form the venous circulation to the arterial circulation as a result of RLS due to a PFO, and if this is related to the size of the PFO, is yet to be elucidated.

In our study, the prevalence of migraines was 50% in the total population and numerically higher in the PFO group compared to the non-PFO group, which is higher than the estimated prevalence of 10% in the general population. These results are in line with the Women’s Ischemia Syndrome Evaluation-Coronary Vascular Dysfunction (WISE-CVD) trial, which showed that 50% of the patients with suspected ANOCA had a history of migraine [17]. Similarly, the prevalence of patients with migraine with aura was numerically higher in the PFO group compared to the non-PFO group (45% vs. 27%, respectively). The association of PFO and migraine has been described in previous studies, as has the association between migraine and ANOCA [5,18,19]. In our study, and perhaps as expected, RLS was numerically more prevalent in patients with CAS and migraine (29%) compared to patients without migraine (17%). Recently, Ravi et al. reported in a small case series the effect of percutaneous PFO closure in patients with CAS, which resulted in an improvement of angina symptoms [20]. This may suggest that RLS may be a contributing mechanism of the occurrence of angina in patients with CAS. However, these observations are subjected to significant observation and/or performance bias. Therefore, the results should be interpreted with caution, and more research is needed to investigate this hypothesis. Interestingly, we found a worse angina SAQ summary score in the patients with PFO, which was statistically significant. Furthermore, numerically worse scores were observed in domains C (angina frequency) and E (Quality of life) of the SAQ (Figure 1C,E), possibly suggesting a modulating function of the PFO due to vasoactive metabolites on angina symptoms. Although percutaneous PFO closure is a low-risk procedure, the effect of PFO closure in patients with CAS and concomitant migraine is currently unknown and warrants further investigation.

Robust evidence of pathophysiological pathways between ANOCA and migraine remains unknown. Consequently, there are several hypotheses as to the possible cause of this association (i.e., dysfunction of the coronary microcirculation and systemic endothelial dysfunction). However, different studies have shown contradictory results with respect to the coronary flow reserve and ICFT in patients with or without migraine [17,21,22]. It must be taken into account that the pathophysiology of CAS is multifactorial (similar to migraine) and not limited to the classical cardiovascular risk factors. Also, mechanisms such as microembolism, inflammation, inflammatory disorders, hypercoagulable stress, platelet dysfunction, metabolite production, and oxidative stress could contribute to angina symptoms [23,24,25]. This could be an explanation as to why the results of the MIDAS Questionnaire are not different between the PFO and non-PFO group (Table 3) as there are different mechanisms contributing to both CAS and migraines causing the same level of disability due to migraines (*p* = 0.631 and *p* = 0.422 for patients with migraine and migraine with aura, respectively). Recently, the LEARNER trial demonstrated an altered oxidative stress in migraine with aura patients compared to the control group after PFO closure [26]. Furthermore, PFO closure resulted in a reduced level of platelet activation and complete remission of migraine in selected patients [26]. Future research may focus on patients with PFO and CAS with concomitant migraine and may include three main components: (1) PFO closure itself; (2) analysis of venous (right atrium) and arterial (left atrium) blood samples (pre and post PFO closure); and (3) patient-reported outcome measures (PROMs). Hopefully, this will provide better insights into the role of different mechanisms and vasoactive metabolites and the effect of PFO closure in patients with CAS. As for the impact on the clinical practice, it is advisable that patients with vasospastic angina, when receiving a TTE, also undergo a bubble contrast study with agitated saline injection. This would allow the cardiologists to track in a registry how many patients with CAS actually have RLS. Additionally, it is important to inquire into the medical history of migraine as we to hypothesize that patients with vasospastic angina and concomitant migraine may have a shared trigger due to RLS [20]. Finally, as a last resort, a PFO closure could be considered for patients who do not respond adequately to medication in selected cases (i.e., patients with ANOCA and concomitant migraine) [20].

### Limitations

This study has several limitations. As this was a single-center study, we were limited to a small sample size. Second, the occurrence of migraine was self-reported by the patients. Although the patients did have migraines in their medical records or histories, this was not always diagnosed by a neurologist. Furthermore, this was an open-label study as not all patients completed the questionnaires before TTE assessment, which could potentially lead to a response bias. Importantly, different dosages of medication could influence the intensity and frequency of angina symptoms and migraine as this is a potential confounder. Finally, we cannot rule out selection bias as only patients with CAS who underwent ICFT were eligible for inclusion.

## 5. Conclusions

This study showed an RLS prevalence due to PFO of 23% in patients with CAS, which was in line with the prevalence of PFO in the general population. The prevalence of RLS in patients with CAS and migraine was higher than in patients without migraine. Further research is needed to investigate the prevalence of PFO in patients with CAS with and without migraine in a larger population. Moreover, the role of vasoactive metabolites, platelet activation, and PFO closure needs to be determined as it may be important to understanding the underlying mechanisms and the suggested role of RLS in patients with both PFO and CAS. Eventually, whether PFO closure could be beneficial in a selected group of patients with CAS and a PFO with concomitant migraine needs to be studied as we need robust clinical trials before any definitive conclusions can be drawn.

## Figures and Tables

**Table 1:** Baseline characteristics of the included patients.

Characteristic	PFO Group (*n* = 11)	Non PFO Group (*n* = 37)	All Patients (*n* = 48)	*p* Value
Age (years), mean (SD)	54 (11)	60 (9)	58 (10)	0.154
Sex at birth, n (%)				0.661
Female	10 (91)	30 (81)	40 (83)	
Male	1 (9.1)	7 (19)	8 (17)	
BMI (kg/m^2^), mean (SD)	23.7 (4.1)	26.4 (3.4)	25.8 (3.7)	0.015
Prior PCI, n (%)	1 (9.1)	8 (22)	9 (19)	0.425
Dyslipidemia, n (%)	3 (27)	12 (32)	15 (31)	1.000
Hypertension, n (%)	2 (18)	18 (49)	20 (42)	0.092
Diabetes mellitus, n (%)	0 (0%)	5 (14)	5 (10)	0.319
Smoking, n (%)				0.080
Never	11 (100)	24 (65)	35 (73)	
Former	0 (0)	11 (30)	11 (23)	
Current	0 (0)	2 (5.4)	2 (4.2)	
Migraine, n (%)	7 (64)	17 (46)	24 (50)	0.303
Migraine with aura, n (%)	5 (45)	10 (27)	15 (31)	0.283
Coronary artery spasm endotype, n (%)				1.000
Epicardial spasm	7 (64)	25 (68)	32 (67)	
Microvascular spasm	4 (36)	12 (32)	16 (33)	
Spasm in epicardial vessel, n (%)				0.803
Single vessel LAD	3 (27)	13 (35)	16 (33)	
Single Vessel Cx	0 (0)	2 (5.0)	2 (4.2)	
Multivessel LAD & Cx	4 (36)	10 (27)	14 (29)	

Abbreviations: BMI = body mass index; CAD = coronary artery disease; LM = left main; LAD = left anterior descending; PFO = patent foramen ovale; PCI = percutaneous coronary intervention; Cx = circumflex artery; RLS = right-to-left shunting.

**Table 2:** Echocardiographic characteristics.

Characteristic	PFO Group (*n* = 11)	Non PFO Group (*n* = 37)	All Patients (*n* = 48)	*p* Value
Grade of RLS, n (%)				<0.001
Grade 0	-	37 (100)	37 (77)	
Grade I	1 (9.1)	-	1 (2.1)	
Grade II	5 (45)	-	5 (10.4)	
Grade III	5 (45)	-	5 (10.4)	
Grade IV	-	-	-	
Atrial septal aneurysm, n (%)	2 (18)	-	2 (4)	
LVEF in %, median (Q1–Q3)	58.0 (52.5–59.5)	60.0 (55.0–63.0)	59.0 (55.0–61.8)	0.093
LVF, n (%)				0.41
Normal function	10 (91)	36 (97)	46 (96)	
Mild dysfunction	1 (9.1)	1 (2.7)	2 (4.2)	
Diastolic function LV, n (%)				1.00
Normal function	11 (100)	34 (92)	45 (94)	
Grade I dysfunction	-	3 (8.1)	3 (6.3)	
RVF, n (%)				
Normal function	11 (100)	37 (100)	48 (100)	

Abbreviations: ASA = atrial septal aneurysm; LVEF = left ventricular ejection fraction; LVF = left ventricular function; RLS = right-to-left shunt; RVF = right ventricular function.

**Table 3:** Results of the patient-reported symptoms from the Migraine Disability Assessment Questionnaire.

Characteristic	PFO Group	Non PFO Group	Total Patients	*p* Value
All included patients	(*n* = 11)	(*n* = 37)	(*n* = 48)	
MIDAS score, median (Q1–Q3)	2 (0–12)	0 (0–9)	0 (0–10)	0.368
MIDAS classification, n (%)				0.601
Little or no disability	7 (64)	22 (59)	29 (60)	
Mild disability	0 (0)	6 (16)	6 (13)	
Moderate disability	2 (18)	5 (14)	7 (15)	
Severe disability	2 (18)	4 (11)	6 (13)	
Patients with migraine	(*n* = 7)	(*n* = 17)	(*n* = 24)	
MIDAS score, median (Q1–Q3)	12 (2–19)	9 (0–16)	10 (2–17)	0.631
MIDAS classification, n (%)				0.799
Little or no disability	3 (43)	6 (35)	9 (38)	
Mild disability	-	3 (18)	3 (13)	
Moderate disability	2 (29)	4 (24)	6 (25)	
Severe disability	2 (29)	4 (24)	6 (25)	
Patients with migraine and aura	(*n* = 5)	(*n* = 10)	(*n* = 15)	
MIDAS score, median (Q1–Q3)	12 (12–25)	11 (1–18)	12 (3–21)	0.422
MIDAS classification, n (%)				1.000
Little or no disability	1 (20)	3 (30)	4 (27)	
Mild disability	-	1 (10)	1 (6.7)	
Moderate disability	2 (40)	3 (30)	5 (33)	
Severe disability	2 (40)	3 (30)	5 (33)	

Abbreviations: MIDAS = Migraine Disability Assessment.

## Data Availability

The data that support the findings of this study are available on request from the corresponding author. The data are not publicly available due to privacy or ethical restrictions.

## Abbreviations

The following abbreviations are used in this manuscript:

ANOCA	Angina with non-obstructed coronary arteries
CAS	Coronary artery spasm
ICFT	Intracoronary function test
MIDAS	Migraine disability assessment
PFO	Patent foramen ovale
PROVA	Prevalence of patent foramen ovale in patients with non-obstructive coronary artery disease
SAQ	Seattle angina questionnaire
TTE	Tansthoracic echocardiography
